# Secular trends in Javanese adult height: the roles of environment and educational attainment

**DOI:** 10.1186/s12889-022-13144-6

**Published:** 2022-04-11

**Authors:** Annang Giri Moelyo, Mei Neni Sitaresmi, Madarina Julia

**Affiliations:** 1 Doctorate program, Faculty of Medicine, Public Health and Nursing, Universitas Gadjah Mada, Yogyakarta, Indonesia; 2 Department of Child Health, Faculty of Medicine/Dr. Moewardi Hospital, Universitas Sebelas Maret, Surakarta, Indonesia; 3 Department of Child Health, Faculty of Medicine, Public Health and Nursing, Universitas Gadjah Mada/Dr. Sardjito Hospital, Yogyakarta, Indonesia

**Keywords:** Adult height, Secular trend, City, Rural, Education, Socioeconomic, Household food expenditure shares

## Abstract

**Background:**

Since its independence in 1945, Indonesia has undergone rapid socioeconomic development. The fastest changes occurred in Java, which is the main island where more than half of the Indonesian population lives.

**Objective:**

This study aimed to analyze the secular trend in the height of adults living in Java born between 1953 and 1995 by comparing their residence (rural, small city, or large city) and considering factors that contribute to adult height.

**Methods:**

The analysis used the following data: birth year, body height, weight, body mass index, sex, educational attainment, share of household food expenditures, and residence of 20- to 40-year-old men and women collected by the Indonesia Family Life Survey (IFLS) waves 1 to 5. Multiple linear regression was conducted to analyze several factors that influence adult height. Significance was set at *p* < 0.05 with a 95% confidence interval (CI).

**Results:**

The study included 30,656 measurements of individuals born between 1953 and 1995 (54.9% female). Positive secular trends (95% CI) were observed for men and women: 1.3 (1.1–1.4) cm and 0.9 (0.8–1.0) cm per decade, respectively. Multiple linear regression analyses showed that, in addition to the year of birth, the adult height of both males and females was independently associated with level of education and share of household food expenditure. Stratifying the data into residence in rural areas, small cities, and large cities showed that education levels influenced the adult height of men and women living in all regions, whereas the influence of birth year and share of household food expenditure differed between areas and genders.

**Conclusions:**

We observed positive secular trends in the height of adults living in Java who were born between 1953 and 1995. The birth year, educational attainment, and share of household food expenditure significantly influenced adult height. A higher education level was consistently associated with taller adult height in both men and women living in rural areas, small cities, and large cities.

**Supplementary Information:**

The online version contains supplementary material available at 10.1186/s12889-022-13144-6.

## Introduction

Later generations are almost always taller than their predecessors; this phenomenon is called a secular trend in height [1]. Every country has grown taller at its own pace and in its own time ⁠[2]. The Dutch, who quickly became the world’s tallest people, have stopped growing taller [3]⁠, whereas most Asian countries continue to grow taller at varying rates [1, 2, 4].

Secular trends in height are influenced by a complex interaction of many factors [5–7]. Environmental conditions, especially socioeconomic development, are usually considered the most important factor influencing secular trends in height [1, 8, 9]. However, socioeconomic prosperity is challenging to measure [10]⁠. Educational level can influence adult height by serving as a proxy for socioeconomic prosperity. More prosperous people typically have better educational opportunities and attainment, and better-educated people are usually wealthier ⁠[8, 9, 11]. In countries that have undergone an economic transition, socioeconomic prosperity is also associated with a shift in the population’s nutritional status; i.e., less underweight, more overweight, and increased mean body mass index [12, 13]. Urbanization, migration, interconnectivity, and neighboring areas (community effect on height) have also contributed to secular trends in height [4, 14].

Indonesia is an archipelago with a wide diversity of ethnicities and languages. Its population has improved rapidly in terms of socioeconomic conditions since its independence in 1945, although vast inequalities yet remain, such as differences in living conditions between urban and rural areas and other inequalities among its islands. These inequalities are mainly related to the availability of infrastructure, especially the ease of transportation and connections. The largest land mass is Java Island, which is home to the Indonesian capital Jakarta, the most populated and prosperous area of Indonesia. More than half of the Indonesian population lives on Java. In addition, Java has several densely populated cities or even metropolitan areas with international connections through their airports or seaports, which have been ports of entry for foreign visitors for many centuries.

Between 1995 and 2015, the population of Java grew from 114 million to 145 million, with a population density ranging from 813 to 15,328 per square kilometer [15]⁠. The respective lowest and highest gross domestic regional product per capita of the Java Island provinces improved from 3.7 and 25.4, respectively, in 1995 to 22.7 and 142.9, respectively, in 2015 (in million IDR) [16]. The percentage of those with low income in Java was 10.8% in 2014 (8.4% urban vs. 14.25% rural), with a national poverty line of 1.13 USD per day [17]. Between 1995 and 2015, the average years of schooling increased from 7.6 to 8.3 years [16].

The Indonesian people are relatively shorter than the rest of the world’s average height, even compared with people from some Southeast Asian countries [18, 19]. This study aimed to analyze the secular trend in height of adults living in Java born between 1953 and 1995. We also analyzed factors contributing to adult height: area of residence and share of household food expenditure as proxies for socioeconomic status, nutritional status, and educational level.

## Methods

The Indonesia Family Life Survey (IFLS) was conducted by the RAND Corporation in collaboration with Universitas Indonesia and Universitas Gadjah Mada. The data are freely available upon request at www.rand.org. The surveys were conducted in five waves: 1993–1994, 1997–1998, 2000, 2007, and 2014 [20–24]. The survey sampling was stratified by province. From a national socioeconomic survey of 60,000 households (the 1993 SUSENAS), enumeration areas were randomly chosen within each province. The survey sample was collected in 13 of the country’s 26 provinces and represented approximately 83% of the Indonesian population. Data from Java Island constituted 59.8% of the total IFLS data [25].

We downloaded the data in October 2019. This study included data from individuals aged 20–40 years who lived in Java. At that age range, we assumed that the participant’s final height had been attained before the shrinking in older age occurred. We collected data on birth year, sex, height, weight, body mass index, place of residence, educational attainment, and the household food expenditure share in relation to total expenditure. Body mass index, as a marker for nutritional status, was defined based on the World Health Organization criteria: underweight (< 18 kg/m^2^), normal weight (18–25 kg/m^2^), overweight (25–30 kg/m^2^), and obese (> 30 kg/m^2^) [26].

The environmental backgrounds were determined by the place of residence, i.e., urban or rural. The rural area was distinguished from the urban area by several characteristics defined by the Indonesian Bureau of Statistics: the size of the area, the population’s size and density, the main occupation of its people, and the availability of public facilities. Furthermore, the urban area was divided into small and large cities based on population density (> 1000 population/km^2^), population size (> 500,000 people), and the presence of ports (airport or seaport) [27].

Educational attainment was defined as the length of education in years. Education levels were classified as basic education (≤9 years of schooling) and high-level education (> 9 years of education) [17]. The monthly household food expenditure share, as a proxy of household economic level, was the monthly food expenditure divided by the total expenditure in the household. The lower their share, the richer the individual’s household [28].

### Statistical analyses

We constructed scatter plots of adult height (cm) on the Y-axis against the year of birth on the X-axis, stratified by sex, using standard deviation (SD). The mean and 2 SD score below and above the mean lines emphasize the trend. Assuming linearity of the secular trend, we estimated the trend using the slope of the linear regression lines, with adjustment of age-associated stature loss, stratified by gender. The secular trend in height was calculated from the linear regression formula (*y* = *az* + *bx* + *c*) (*y* = adult height in cm after *x* years; *a* = the height changes in cm per year of age; *z* = age in years; *b* = the secular trend in adult height per year in cm/year; *x* = year differences [the birth year minus 1953]; *c* = a constant); the year 1953 was the minimum birth year for our subjects. The secular trend was then converted to centimeters/decade.

We used a one-way analysis of variance (ANOVA) followed by the Bonferroni post hoc test to compare the heights of the populations of rural areas, small cities, and large cities in every five-year interval of birth year stratified by gender. For example, birth year “1955” meant those who were born between July 1952 and June 1957; “1960” meant July 1957 to June 1962, and so forth. Multiple linear regressions stratified by gender and residence were conducted to assess the interaction between factors influencing adult height, such as birth year, educational level, nutritional status, and household economic level. Statistical analyses were performed using STATA/MP 14.0 with a 95% confidence interval (CI).

## Results

The study included 30,656 (54.7%) measurements of individuals who had complete data out of a total 56,085 people aged 20–40 years in the IFLS waves 1, 2, 3, 4, and 5. The proportion of women was between 52.5 and 60.4%, and those living in rural areas, small cities, and large cities remained relatively constant, approximately 66.4–72.3%, 6.1–7.1%, and 21.3–26.5%, respectively. The proportion of those who had finished at least 9 years of schooling steadily increased, with 40.1% in wave 3 and 52.7% in wave 5. No subjects had finished more than 9 years of schooling in waves 1 and 2. The median (Q1; Q3) share of household food expenditure also decreased steadily, with 0.23 (0.12; 0.39) in wave 1 to 0.12 (0.07; 0.22) in wave 5 (Supplementary Table 1).

Table 1 shows the means (SD) of adult height in every five-year interval of birth year. The one-way ANOVA showed that, except for men born in 1995 and women born in 1955 and 1995, there were significant differences between groups according to area of residence. Prior to those born in 1990, both men and women living in urban areas, especially those living in large cities, were always significantly taller than those living in rural areas.

**Table 1:** Javanese adult height among total, rural, small cities and large cities

Birthyear	total			rural			small cities			large cities			
	n	Means of height	SD	n	Means of height	SD	n	Means of height	SD	n	Means of height	SD	p
Men													
1955	267	161.2	5.8	175	160.6	5.8	29	161.4	4.5	63	162.9	6.2	0.031 ^a^
1960	1123	161.4	5.7	814	161.0	5.6	63	162.9	5.8	246	162.5	5.9	0.000 ^a,b^
1965	1540	162.4	5.8	1075	162.1	5.8	91	162.9	6.2	374	163.3	5.7	0.002 ^a^
1970	2257	162.6	5.8	1572	162.3	5.9	162	163.9	5.6	523	163.4	5.6	0.000 ^a,b^
1975	2801	163.0	6.1	1923	162.5	6.1	169	163.6	5.9	709	164.3	5.8	0.000 ^a^
1980	2771	164.0	6.0	1937	163.6	5.8	184	163.6	6.3	650	165.2	6.1	0.000 ^a,c^
1985	1800	164.7	5.9	1273	164.4	5.9	101	164.9	6.1	426	165.3	5.8	0.031 ^a^
1990	881	164.9	6.1	627	164.5	6.0	55	166.6	5.9	199	165.9	6.4	0.002 ^a,b^
1995	389	165.8	6.2	264	165.4	6.2	29	166.7	7.1	96	166.6	5.9	0.172
Women													
1955	333	150.2	5.4	210	150.3	5.4	24	148.9	5.5	99	150.5	5.3	0.413
1960	1352	150.7	5.1	949	150.3	5.0	84	152.2	4.9	319	151.3	5.2	0.000 ^a,b^
1965	2038	150.6	5.1	1392	150.4	5.1	150	151.0	4.2	496	151.0	5.3	0.046
1970	2750	151.0	5.3	1883	150.7	5.2	204	151.8	5.3	663	151.7	5.3	0.000 ^a,b^
1975	3020	151.1	5.4	2066	150.7	5.2	185	152.0	5.3	769	152.0	5.7	0.000 ^a,b^
1980	3338	152.0	5.5	2392	151.6	5.5	213	152.7	5.4	733	152.9	5.4	0.000 ^a,b^
1985	2307	152.2	5.3	1632	151.8	5.2	153	152.3	5.0	522	153.2	5.3	0.000 ^a^
1990	1195	152.7	5.3	872	152.2	5.2	72	153.5	4.9	251	154.0	5.3	0.000 ^a^
1995	494	153.6	5.3	348	153.3	5.1	29	154.2	7.7	117	154.3	5.2	0.157

Posthoc analysis (Bonferonni test); ^a^: significant between large city vs rural; ^b^: significant between small city vs rural; ^c^: significant between large city vs small city

**Fig. 1:**
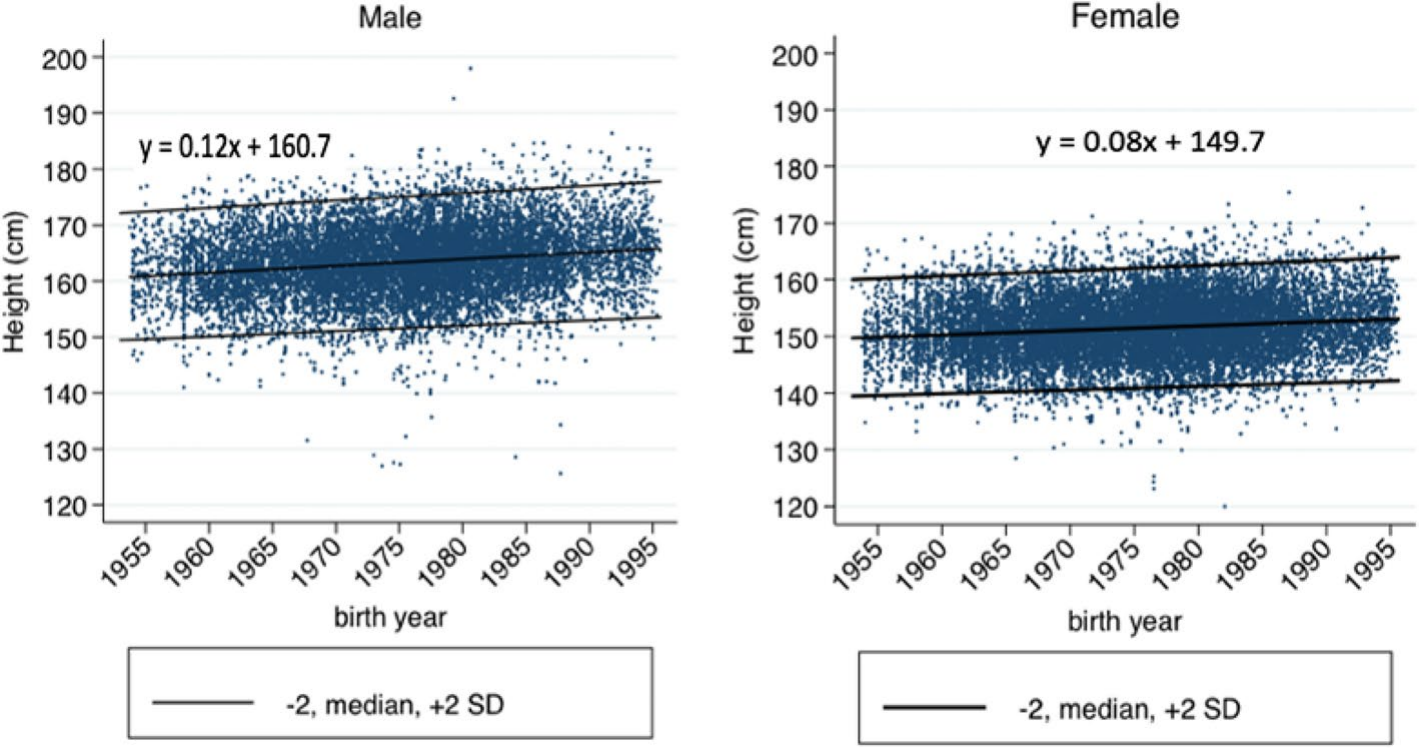
Scatter plot of secular trend in adult male (**A**) and female (**B**) height. (y = ax + b; y = adult height in cm after x years; a = the secular trend in adult height per year in cm/year; x = year differences [the birth year minus 1953]; b = a constant; stratified by gender, without adjusting for age-associated stature loss)

We observed positive secular trends in adult height for both men and women, whether in total or in rural areas, small cities, or large cities (Fig. 1). The linear regression formulas for adult height were *y* = 0.02*z* + 0.13*x* + 160.0 for males and *y* = 0.04*z* + 0.09*x* + 148.3 for females (*y* = height attained in cm; *z* = age; *x* = year of birth, beginning from the year 1953 as 0). The secular trend in adult height, based on the formulas, increased 1.3 cm and 0.9 cm/decade for men and women, respectively. After adjusting for various other factors, the adult height increases (95% CI) for men and women were 0.6 (0.5–0.8) and 0.5 (0.4–0.6) cm/decade, respectively (Table 2). Table 2 also shows that year of birth, level of education, and share of household food expenditure were significantly and independently associated with adult height both in men and women. Nutritional status had a significant negative association with adult height in women but not in men. Obese women had a shorter height than underweight women.

**Table 2:** Univariate and multivariate analysis of Javanese adult height

	Univariate			Multivariate		
	Coeff.	95% CI	const	Coeff.	95% CI	const.
Men					*(n = 13,829)*	
age	(−0.11)	(− 0.13)-(− 0.09)	166.6	(− 0.03)	(− 0.05)-(− 0.01)*	
birth year	0.12	0.11–0.13*	160.7	0.06	0.05–0.08*	
high education (vs basic education/reff)	2.90	2.70–3.10*	162.2	2.35	2.13–2.57*	
nutritional status (underweight/reff)						
normoweight	(−0.82)	(−1.10)-(− 0.53)*		(− 0.58)	(− 0.86)-(− 0.30)*	
overweight	0.13	(− 0.25)-(0.51)*		(− 0.15)	(− 0.53)-0.23	
obese	0.74	0.34–1.45*	163.9	(−0.101)	(−0.79)-0.59	
household food expenditure share	(−3.53)	(−4.15)-(− 2.90)*	164.0	(−1.93)	(− 2.55)-(− 1.32)*	
adj. r2				0.07		44.47
Women					*(n = 16,827)*	
age	(−0.55)	(−0.07)-(−0.41)	153.1	0.01	(−0.01)-(0.03)	
birth year	0.08	0.07–0.09*	(−3.3)	0.05	0.04–0.06*	
high education (vs basic education/reff)	2.22	2.05–2.39*	150.8	1.80	1.63–2.00*	
nutritional status (underweight/reff)						
normoweight	(−0.43)	(−0.69)-(−0.16)*		(−0.38)	(−0.64)-(− 0.11)*	
overweight	(−0.46)	(− 0.76)-(− 0.15)*		(−0.49)	(− 0.79)-(− 0.18)*	
obese	(− 0.29)	(− 0.69)-0.10	151.9	(− 0.54)	(−0.94)-(− 0.15)*	
household food expenditure share	(−2.54)	(−3.05)-(−2.04)*	152.0	(−1.45)	(−1.96)-(− 0.95)*	
adj. r2				0.04		59.33

*p < 0.05

Before adjustment, age, birth year, and education were consistently associated with adult height in large cities, small cities, and rural areas and in men and women (*p* < 0.05). After adjustment, stratified by place of residence, the secular trends (95% CI) of men in rural areas, small cities, and large cities were 0.8 (0.6 to 0.9), 0.4 (− 0.2 to 1.0), and 0.5 (0.2 to 0.9) cm/decade, respectively. The adult height secular trends (95% CI) of women in rural areas, small cities, and large cities were 0.6 (0.4 to 0.7), 0.2 (− 0.2 to 0.7), and 0.6 (0.3 to 0.8) cm/decade, respectively (Table 3).

**Table 3:** Javanese adult height stratified by place of residence

	Men				Women			
	Unadjusted		Adjusted		Unadjusted		Adjusted	
	Coeff	95%CI	Coeff.	95% CI	Coeff	95%CI	Coeff.	95% CI
Large cities				*(n = 3286)*				*(n = 3969)*
age	(−0.10)	(−0.14)-(− 0.06)*	(− 0.04)	(−0.08)-0.01	(− 0.08)	(− 0.11)-(− 0.05)*	0.01	(−0.03)-0.05
birth year	0.11	0.09–0.14*	0.05	0.02–0.09*	0.10	0.08–0.12*	0.06	0.03–0.08*
high education (vs basic education/reff)	2.37	1.96–2.77*	1.89	1.44–2.34*	2.26	1.93–2.60*	1.78	1.39–2.16*
nutritional status (underweight/reff)								
normoweight	(−0.86)	(− 1.42)-(− 0.30)*	(−0.69)	(− 1.24)-(− 0.14)*	(−0.40)	(− 0.97)-0.18	(− 0.30)	(−0.86)-0.27
overweight	(−0.33)	(− 1.02)-0.36	(− 0.31)	(− 1.00)-0.39	(− 0.93)	(− 1.57)-(− 0.28)*	(−0.78)	(−1.43)-(− 0.14)*
obese	1.23	0.07–2.39*	0.84	(−0.31)-1.98	(−0.04)	(− 0.83)-0.75	(− 0.10)	(−0.89)-0.69
household food expenditure share	(−1.48)	(− 3.03)-0.06	(−0.73)	(−2.25)-0.79	(− 2.17)	(− 3.45)-(− 0.89)*	(−1.38)	(− 2.64)-(− 0.12)*
cons.			163.79	161.92–165.67*			150.62	149.01–152.22*
adj. r2			0.05				0.05	
Small cities				*(n = 883)*				*(n = 1114)*
age	(−0.11)	(−0.18)-(− 0.04)*	(− 0.05)	(− 0.15)-0.04	(− 0.07)	(−0.12)-(− 0.01)*	(−0.02)	(− 0.09)-0.06
birth year	0.11	0.06–0.15*	0.04	(−0.02)-0.10	0.07	0.04–0.10*	0.02	(−0.02)-0.07
high education (vs basic education/reff)	2.50	1.72–3.29*	2.12	1.25–3.00*	1.65	1.04–2.25*	1.43	0.73–2.13*
nutritional status (underweight/reff)								
normoweight	(−0.67)	(−1.91)-0.57	(−0.71)	(−1.93)-0.51	0.17	(−0.95)-1.29	(− 0.00)	(− 1.11)-1.10
overweight	(− 0.07)	(− 1.64)-1.50	(− 0.32)	(−1.89)-1.26	(− 0.69)	(− 1.92)-0.54	(−0.69)	(−1.92)-0.54
obese	1.19	(−1.36)-3.74	0.46	(−2.06)-2.99	(−0.65)	(− 2.13)-0.84	(− 0.91)	(− 2.39)-0.57
household food expenditure share	(− 0.36)	(− 3.09)-2.37	0.21	(− 2.52)-2.94	(− 3.21)	(−5.33)-(− 1.09)*	(− 3.17)	(−5.30)-(− 1.04)*
cons.			163.91	160.05–167.05*			152.16	149.11–155.20*
adj. r2			0.05				0.04	
Rural area				*(n = 9660)*				*(n = 11,744)*
age	(−0.10)	(−0.12)-(− 0.08)*	(− 0.01)	(− 0.03)-(0.02)	(− 0.04)	(−0.06)-(− 0.03)*	0.03	0.01–0.05*
birth year	0.12	0.11–0.14*	0.08	0.06–0.09*	0.07	0.06–0.08*	0.06	0.04–0.07*
high education (vs basic education/reff)	2.94	2.69–3.19*	2.33	2.06–2.60*	2.04	1.83–2.26*	1.62	1.39–1.85*
nutritional status (underweight/reff)								
normoweight	(−0.71)	(− 1.05)-(− 0.36)*	(−0.45)	(− 0.78)-(− 0.11)*	(−0.50)	(− 0.81)-(− 0.18)*	(−0.45)	(− 0.76)-(− 0.14)*
overweight	0.24	(− 0.23)-0.72	(− 0.13)	(−0.60)-0.34	(− 0.31)	(− 0.67)-0.05	(−0.41)	(− 0.78)-(− 0.05)*
obese	(− 0.05)	(− 1.00)-0.90	(− 1.03)	(− 1.95)-(− 0.10)*	(−0.51)	(− 1.00)-(− 0.03)*	(−0.82)	(− 1.31)-(− 0.34)*
household food expenditure share	(−3.54)	(−4.25)-(− 2.83)*	(− 1.91)	(− 2.61)-(− 1.21)*	(−2.09)	(− 2.66)-(− 1.51)*	(− 0.99)	(− 1.57)-(− 0.41)*
cons.			161.43	160.29–162.57*			149.20	148.28–150.12*
adj. r2			0.07				0.04	

*p < 0.05

Table 3 shows that levels of education after adjustment were consistently independently associated with adult height in both men and women in rural areas, small cities, and large cities. However, in rural areas and small cities, the beta coefficient of the educational level was much higher in men than in women. Only in large cities were the beta coefficients of the educational level comparable.

Share of household food expenditure, as a proxy of household economy, were only consistently shown to be independently associated with the adult height of women before and after adjustment. The household economy was associated only with the adult height of rural men, not of men in other areas.

## Discussion

Our study revealed positive secular trends in adult height for men and women in Java, including in large cities, small cities, and rural areas. The average secular trends showed that men had secular trends higher than women (1.3 vs. 0.9 cm/decade). Previous studies have mentioned that changes in the environment affect growth in boys more than in girls [5]. The development of Java, as the main island in Indonesia, the high density of the island, and its mobility could influence this trend not only for males but also for females at different rates. Other studies have shown similar results.

A previous study on the secular trend in Javanese adult height revealed an increase of 3.6 cm in 60 years for men, smaller than that of the Japanese during the same time frame, resulting in the Javanese male being shorter than the Japanese male from 1992 to 1994 [29]. A study in Shandong, China, on 18-year-old participants showed positive secular trends from 1956 to 2000, with rates of 1.75 cm and 1.07 cm per decade for males and females [30]⁠. Another study of nine cities in China revealed increments of 1.3 cm and 0.8 cm per decade in 18-year-old men and women [31]⁠. Genetic factors are known to be among the determinants of body height; however, genetic roles in secular trends in height are less important than environmental factors [1, 6, 32]. Genetic factors also play a role in sexual dysmorphism; males have been 8% taller than females over the last 110 years [5].

Roche reported that the secular changes in stature differ from country to country [7]. Contrary to data from countries in Asia, European reports on flattened secular trends in body height are becoming more frequent. The Dutch, who are considered the tallest people globally, had the same body height, which was 183.8 cm, as 21-year-olds in 1997 and 2009 [3]. However, Mexico’s adult men and women in 2000 were taller than those in 1978⁠ [12]. The final average adult height of men (162.8 cm) and women (153.3 cm) living in Java was above the average Indonesian height. Our study also showed, however, that Javanese people were shorter than the people of Bali, which is the province with the tallest population in Indonesia (165.7 vs. 167.1 cm in men; 153.6 vs. 157.1 cm in women) [18]. Although Java is the main island of Indonesia, the fact that the people of Bali are the tallest needs another explanation. A previous study had shown that the data on Indonesian adult height began to be collected in 1870. From 1870 to 1940, the secular trend was not clearly considered as an interaction between malnutrition and the disease environment, and the trend became more pronounced since 1945 after national independence. This pattern is still increasing, in contrast to the United States, which has reached a plateau [2, 7, 33].

Population density, geography, citizen mobilization, and intercity connectivity have been other factors found to affect adult height [4, 33–35]. Our study showed height differences among rural, small, and large cities for every 5 years of birth (Table 1). A post hoc analysis showed that individuals who lived in large cities were significantly taller than those in rural areas. The height differences between small and large cities were not pronounced, nor were those between small cities and rural areas. Well-connected infrastructure between cities (and rural areas) on Java might explain why the difference was not obvious. The development of transportation on Java Island creates better ease of movement between large cities, small cities, and rural areas. These conditions can be seen in large cities as the main place influenced by modern development. The population density in the large cities, the presence of airports and seaports that make it possible to be more mobile, the economic advantage, and better standards of living might explain why the people who lived in large cities, either male or female, were taller than those in rural areas. Public facilities such as schools and health facilities are also accessed more easily in large cities, although structural improvements occurred across all provinces in Indonesia over the period from 2005 to 2018 [36]. Better health facilities and nutritional supply systems in urban areas contribute to preventing stunted growth in children. Urban children therefore potentially had better growth, thus resulting in taller stature as adults [25]. The infant mortality rate in Indonesia has generally decreased over time, in large part due to improvements in the national health system. These improvements could also help explain the secular trend in adult height in Indonesia (Supplementary Fig. 1).

Demographic change has taken place in Java. The island of Java became a mega urban island between 2000 and 2010 [37]. As noted in the Supplementary Table, the proportion of individuals studied in urban areas has decreased compared with a previous report [37]. Additionally, in a previous report, greatly expanding connectivity between large cities was noted, whereas small and medium-sized cities as centers of socioeconomic activity tended to stagnate [37]. This stagnation could explain why the secular trend in height in a small city is increasing less than that in a large city. Interestingly, from our data, individuals living in rural areas had secular trends in height comparable with those living in large cities. Our data revealed that factors such as the level of education, economic status, and nutritional status were more pronounced, affecting adult height in rural areas. Other factors that might affect adult height, such as gender, educational level, economic status, and nutritional status, were adjusted for in the multivariate analysis. Previous studies had reported that the height of children and adults differed according to region (demographics) and social class [1, 5, 6]. The secular trend was affected by quality of life, urbanization, modernization, water and electricity access, nutrition, and health care [38].

Notably, males and females living in large cities had comparable beta coefficients for several factors (birth year, educational attainment, household economic level, and nutritional status). Males and females had similar potential for their adult height, although sexual dysmorphism still existed. A previous study in Thailand had found no difference in patterns of secular trends between males and females when comparing urban and rural residents. However, the final adult height indicated that the urban population was taller [9]. Koziel et al. found that upper-class fathers with downward social mobility had shorter stature up to the third generation than lower-class fathers with social mobility (having received secondary or university education) [35]. Research performed on Polish university students showed that economic transformations increased the trend in body height [39].

Previous studies have shown that the secular trend in stature occurred at different rates within countries across all socioeconomic groups [7]. Our study showed that the influence of household economic level in large and small cities was more evident in women than men. This finding indicated that factors other than economic level influenced the height of male adults who lived in urban areas. It is possible that males are generally more mobile, more connected, and more competitive with each other. Indonesian data showed that the gender parity index, as an index of gender inequality, was still more favorable for men than for women, particularly in employment, even though the education gap has narrowed [40]. Our study revealed that the household economic level was comparable between women and men in rural areas and in small and large cities. The present data showed that women living in rural areas predominantly had lower levels of high-level education (26.7%) than those living in small cities (52.5%) and large cities (45.1%). Marital status was not available in our data.

The final adult height in large cities, small cities, and rural areas was affected the most by education level compared with other factors (economy/share of household food expenditure, secular trend/birth year, nutritional status/obesity). The role of education in body height has been evident in previous studies. One study in Korea found that individuals with high education levels were 4 to 5 cm taller than those with no or only elementary education. This difference suggests the influence of educational status as a fundamental factor in South Korea’s height data [11]⁠. Male workers in Chile with a higher level of education (university vs. primary/secondary education) were found to be taller [41]. Research from Poland highlighted the importance of education as a major confounder of body height, even though the fathers’ social status had a persistent effect on their children’s height [35]⁠. In our study, the difference in adult height between high-level and basic education was 2.4 cm in men and 1.8 cm in women. A previous study had noted that wealthier people usually had better educational opportunities and attainment. Therefore, adult height reflects educational attainment [42]. Furthermore, better educated people were typically wealthier⁠ [9]. Based on evidence that adult height is a mirror of the societal condition, one study concluded that higher educational attainment is one proxy of the wealthy in the community and hence positively influences adult height [6].

Given that the predictors in our regression models only explain approximately 4–7% of the variability in adult height, there must be other factors that contribute to it, such as pubertal timing, obesity, and demographic transition. Pubertal timing has been shown to be an important factor influencing secular trends [7]. A previous study had shown that the secular trend in height in Korea was greater than that in Japan because of different pubertal timing [43]. A similar study from China also mentioned the importance of sexual maturation on secular trends in height [31]. Unfortunately, we were not able to assess the influence of pubertal timing in our dataset. Further studies to assess the association between pubertal timing and secular trends in the final height of the Indonesian population are needed.

Obesity in childhood has been reported to influence pubertal timing. Children with obesity tend to have an earlier puberty onset as well as less adolescent height gain [44]. We observed that obesity, especially among females in rural areas, was negatively associated with adult height. The prevalence of obesity in females was larger than that in males in rural areas, small cities, and large cities. Our data also showed a positive secular trend in body mass index in women but not in men. The prevalence of obesity was comparable in our data between those with low and high educational attainment.

### Limitations and further study

A limitation of our study was the proportion of respondents; the proportion in small cities was not comparable with that in large cities and rural areas, although there was a normal distribution of adult height overall. Furthermore, given this was a cross-sectional study, we cannot conclude that there is a causal connection between level of education and final height. Further study is needed to analyze the correlation between population mobility-interconnectivity and body height. There are various races and ethnicities among the people living on the island of Java. The genetic or ethnic backgrounds of the studied individuals who lived on Java Island were not available. Therefore, we could not analyze genetic or ethnic factors. Further studies are also needed to analyze the secular trend in height among Indonesian children as reported by Cole, which stated that adult height gains were achieved as early as 2 years of age [5].

## Conclusions

Between 1955 and 1995, there was a positive secular trend in height among adults living in Java, either in a large city, small city, or in rural areas. Education level, place of residence (large city, small city, or rural area), share of household food expenditure, and birth year influenced adult height in both men and women. A high education level was the most influential factor for adult height. People who lived in a large city were taller than those in small cities or rural areas. Men and women who lived in a large city had comparable influencing factors for adult height.

## Supplementary Information

**Additional file 1: Supplementary Table 1**. Baseline characteristic of subjects.

**Additional file 2: Supplementary Figure 1**. Infant mortality rate in Indonesia from 1955 to 1995 (downloaded from https://www.macrotrends.net/countries/IDN/indonesia/infant-mortality-rate in January 24th, 2022).

## Data Availability

The datasets generated and/or analyzed during the current study are available at 10.6084/m9.figshare.16632799.
