# Supplementary material for: Secular trends in Javanese adult height: the roles of environment and educational attainment
Source: BMC Public Health. 2022 Apr 11;22:712. doi: 10.1186/s12889-022-13144-6 (PMC8996584; doi:10.1186/s12889-022-13144-6)
Supplement: Supplementary file 1 — Additional file 1: Supplementary Table 1. Baseline characteristic of subjects. [file 12889_2022_13144_MOESM1_ESM.docx]

Supplementary Table 1. Baseline characteristic of subjects

|  | Wave 1  (year 1993-1994)  n=2,956 | | Wave 2  (year 1997-1998)  n=4,678 | | Wave 3  (year 2000)  n=6,808 | | Wave 4  (year 2007)  n=7,846 | | Wave 5  (year 2014)  n=8,368 | | Total  n=30,656 | |
| --- | --- | --- | --- | --- | --- | --- | --- | --- | --- | --- | --- | --- |
|  | n | % | n | % | n | % | n | % | n | % | n | % |
| Gender |  |  |  |  |  |  |  |  |  |  |  |  |
| Female | 1,784 | 60.35 | 2,650 | 56.65 | 3,576 | 52.53 | 4,185 | 53.34 | 4,632 | 55.35 | 16,827 | 54.89 |
| Male | 1,172 | 39.65 | 2,028 | 43.35 | 3,232 | 47.47 | 3,661 | 46.66 | 3,736 | 44.65 | 13,829 | 45.11 |
| Place of residence |  |  |  |  |  |  |  |  |  |  |  |  |
| Rural | 1,980 | 66.98 | 3,235 | 69.15 | 4,523 | 66.44 | 5,616 | 71.58 | 6,050 | 72.3 | 21,404 | 69.82 |
| Small cities | 194 | 6.56 | 305 | 6.52 | 484 | 7.11 | 477 | 6.08 | 537 | 6.42 | 1,997 | 6.51 |
| Large cities | 782 | 26.45 | 1,138 | 24.33 | 1,801 | 26.45 | 1,753 | 22.34 | 1,781 | 21.28 | 7,255 | 23.67 |
| Education level |  |  |  |  |  |  |  |  |  |  |  |  |
| Basic education (<=9 year) | 2,956 | 100 | 4,678 | 100 | 4,076 | 59.87 | 4,104 | 52.31 | 3,958 | 47.30 | 19,772 | 64.50 |
| High education (>9 year) | 0 | 0 | 0 | 0 | 2,732 | 40.13 | 3,742 | 47.69 | 4,410 | 52.70 | 10,884 | 35.50 |
| Nutritional status |  |  |  |  |  |  |  |  |  |  |  |  |
| Underweight | 346 | 11.71 | 614 | 13.13 | 1,027 | 15.09 | 959 | 12.22 | 884 | 10.56 | 3,830 | 12.49 |
| Normoweight | 2,141 | 72.43 | 3,391 | 72.49 | 4,691 | 68.90 | 5,076 | 64.70 | 4,729 | 56.51 | 20,028 | 65.33 |
| Overweight | 406 | 13.73 | 563 | 12.04 | 921 | 13.53 | 1,435 | 18.29 | 1,992 | 23.80 | 5,317 | 17.34 |
| Obese | 63 | 2.13 | 110 | 2.35 | 169 | 2.48 | 376 | 4.79 | 763 | 9.12 | 1,481 | 4.83 |
| Household food expenditure share |  |  |  |  |  |  |  |  |  |  |  |  |
| Q1 | 0.122 |  | 0.052 |  | 0.092 |  | 0.077 |  | 0.067 |  | 0.074 |  |
| median | 0.227 |  | 0.105 |  | 0.172 |  | 0.140 |  | 0.124 |  | 0.142 |  |
| Q3 | 0.387 |  | 0.205 |  | 0.314 |  | 0.239 |  | 0.217 |  | 0.259 |  |
