# Supplementary material for: Secular trends in Javanese adult height: the roles of environment and educational attainment
Source: BMC Public Health. 2022 Apr 11;22:712. doi: 10.1186/s12889-022-13144-6 (PMC8996584; doi:10.1186/s12889-022-13144-6)
Supplement: Supplementary file 2 — Additional file 2: Supplementary Figure 1. Infant mortality rate in Indonesia from 1955 to 1995 (downloaded from https://www.macrotrends.net/countries/IDN/indonesia/infant-mortality-rate in January 24th, 2022). [file 12889_2022_13144_MOESM2_ESM.docx]

**Supplementary figure**


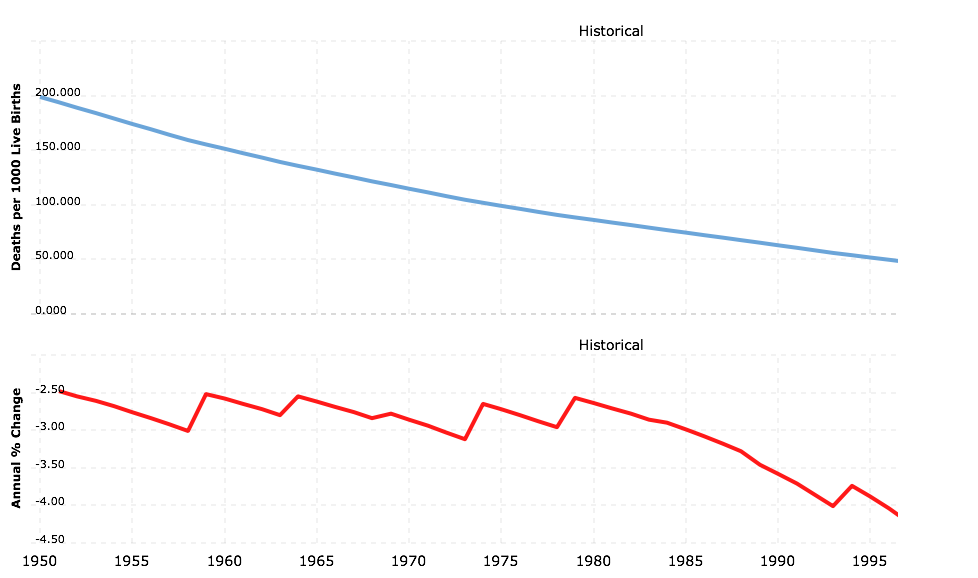


Supplementary Fig.1. Infant mortality rate in Indonesia from 1955-1995 (downloaded from <https://www.macrotrends.net/countries/IDN/indonesia/infant-mortality-rate> in January 24th, 2022)
